# Extracellular cystatin SN and cathepsin B prevent cellular senescence by inhibiting abnormal glycogen accumulation

**DOI:** 10.1038/cddis.2017.153

**Published:** 2017-04-06

**Authors:** Sang-Seok Oh, Soojong Park, Ki-Won Lee, Hamadi Madhi, Sae Gwang Park, Hee Gu Lee, Yong-Yeon Cho, Jiyun Yoo, Kwang Dong Kim

**Affiliations:** 1 Division of Applied Life Science (BK21 Plus), Gyeongsang National University, Jinju, Republic of Korea; 2 Division of Life Science, Gyeongsang National University, Jinju, Republic of Korea; 3 PMBBRC, Gyeongsang National University, Jinju, Republic of Korea; 4 Department of Microbiology, College of Medicine, Inje University, Busan, Republic of Korea; 5 Immunotherapy Convergence Research Center, Korea Research Institute of Bioscience and Biotechnology, Daejeon, Republic of Korea; 6 College of Pharmacy, The Catholic University of Korea, Bucheon, Republic of Korea

## Abstract

Cystatin SN (CST1), a known inhibitor of cathepsin B (CatB), has important roles in tumor development. Paradoxically, CatB is a member of the cysteine cathepsin family that acts in cellular processes, such as tumor development and invasion. However, the relationship between CST1 and CatB, and their roles in tumor development are poorly understood. In this study, we observed that the knockdown of CST1 induced the activity of senescence-associated *β*-galactosidase, a marker of cellular senescence, and expression of senescence-associated secretory phenotype genes, including *interleukin-6* and *chemokine (C-C motif) ligand 20,* in MDA-MB-231 and SW480 cancer cells. Furthermore, CST1 knockdown decreased extracellular CatB activity, and direct CatB inhibition, using specific inhibitors or shCatB, induced cellular senescence. Reconstitution of CST1 restored CatB activity and inhibited cellular senescence in CST1 knockdown cells. CST1 knockdown or CatB inhibition increased glycogen synthase (GS) kinase 3*β* phosphorylation at serine 9, resulting in the activation of GS and the induction of glycogen accumulation associated with cellular senescence. Importantly, CST1 knockdown suppressed cancer cell proliferation, soft agar colony growth and tumor growth in a xenograft model. These results indicate that CST1-mediated extracellular CatB activity enhances tumor development by preventing cellular senescence. Our findings suggest that antagonists of CST1 or inhibitors of CatB are potential anticancer agents.

Cysteine cathepsins perform various functions, including the processing of proteins during antigen presentation, bone modeling and epidermal homeostasis.^1^ Previous reports have shown that the activation of extracellular cathepsins such as cathepsin B (CatB) has an important role in the degradation of extracellular matrix proteins, including collagen, laminin and fibronectin, facilitating tumor metastasis through the remodeling of the extracellular environment.^2, 3, 4^ Moreover, CatB promotes the proliferation, invasion and metastasis of some tumor cells.^3, 4, 5, 6^ The proteolytic activities of CatB are negatively regulated by specific inhibitory proteins belonging to the type 2 cystatin family.^7^ Cystatin SN (CST1), encoded by *CST1,* is a member of the type 2 cystatin family, and the induction of CST1 expression is associated with tumorigenesis, increased cancer cell proliferation and invasion, and tumor recurrence.^8, 9, 10, 11^ As CatB is a functional protease and CST1 is its inhibitor, it is highly paradoxical that both of them contribute to tumorigenesis. To date, the underlying relationship between CST1 and CatB, and their roles in tumor development, remains poorly understood.

Replicative cellular senescence features a permanent cell cycle arrest, resulting in limited cell proliferation.^12^ Repeated DNA replication during normal cell proliferation contributes to the shortening of telomeres, which causes cell cycle arrest and genomic instability.^13, 14^ Premature senescence, an accelerated senescence phenotype, can be induced by various stresses such as oxidative stress, ionizing radiation^15, 16^ and anticancer chemotherapy.^17, 18^ Senescent cells exhibit a diverse range of common characteristics, including the arrest of the cell cycle, activation of tumor-suppressor networks,^19, 20^ morphologic changes,^21, 22^ induction of senescence-associated *β*-galactosidase (SA-*β*-gal) activity^23^ and adoption of a senescence-associated secretory phenotype (SASP).^24^ Generally, cancer cells avoid cellular senescence by developing gene during carcinogenesis and malignant cell transformation.^25^ Thus, cellular senescence has been considered a tumor-suppressive mechanism that inhibits the uncontrolled proliferation of cancer cells.^26, 27^

In this study, we observed that CST1 knockdown induced cellular senescence and decreased extracellular CatB activity in MDA-MB-231 and SW480 cancer cell lines. We hypothesized that the relationship between CST1 and CatB contributed to tumorigenesis via the prevention of cellular senescence. Moreover, we further investigated the molecular mechanisms underlying CST1-mediated prevention against cellular senescence in cancer cells.

## Results

### Knockdown of CST1 induces cellular senescence

**Figure 1:**
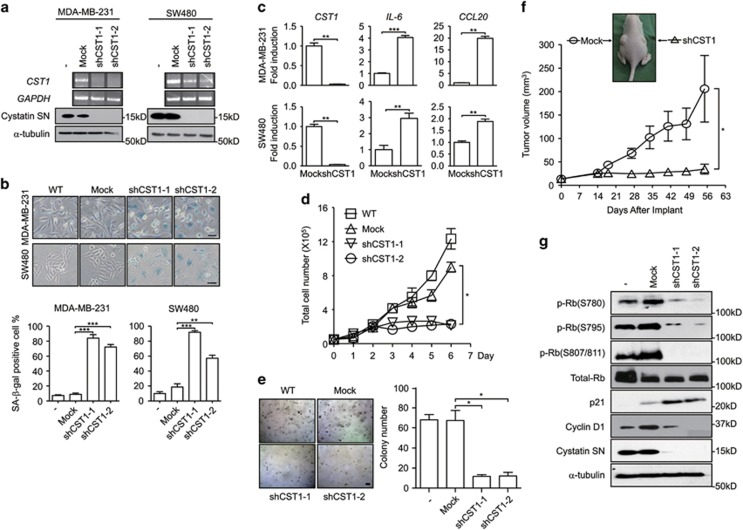
CST1 is required for cell proliferation and colony growth *in vitro* and *in vivo*. The following assays were performed in human cancer MDA-MB-231 and SW480 cells transduced with a control shRNA (mock) or an shRNA for CST1. (**a**) The efficiency of CST1 knockdown was confirmed by reverse transcription PCR (upper) and western blotting (lower). (**b**) Representative photographs of SA-*β*-gal staining (upper). Senescence under the indicated condition was quantified as the percentage of SA-*β*-gal-positive cells (lower). Scale bars, 50 *μ*m. (**c**) The expression of SASP genes induced by CST1 knockdown. RNAs were extracted from cells, and real-time PCR analysis was performed to quantify the transcripts of *CST1*, *IL-6* and *chemokine ligand 20* (CCL20). (**d**) Analysis of anchorage-dependent cell proliferation. Cells were seeded on to six-well plates (5 × 10^4^ cells per well) and cultured for the indicated number of days. The number of cells was counted using a hemocytometer. One-way ANOVA was used for statistical analysis (*P<0.05). (**e**) Analysis of anchorage-independent cell proliferation. Cells were seeded on to complete DMEM containing agarose (1 × 10^5^ cells per well) and cultured for 12 days. Formed colonies were stained with 0.005% crystal violet and quantified under a light microscope. Scale bar, 10 *μ*m. (**f**) Analysis of tumor growth in the xenograft model. MDA-MB-231 cells transduced with a control shRNA or sh*CST1* (1 × 10^6^ cells) were subcutaneously implanted into the left or right flanks of athymic nude mice (*n*=8). Tumor volume was measured on the indicated days. One-way ANOVA was used for statistical analysis (*P<0.05). (**g**) Protein expression of p-Rb, total Rb, p21, cyclin D1 or CST1 was evaluated by western blotting. *P<0.05; **P<0.01; ***P<0.001 by Student's *t*-test

To investigate the role of CST1 in tumorigenesis, we screened human colon and breast cancer cell lines and discovered that CST1 was highly expressed in and was secreted into the culture medium by SW480 and MDA-MB-231 cells (Supplementary Figure 1). In SW480 colon cancer and MDA-MB-231 breast cancer cells, CST1 knockdown using the pLenti expression system containing a short hairpin RNA (shRNA) for CST1 (shCST1; Figure 1a) induced striking morphologic changes, including an enlarged nucleus and flattened cytoplasm, and increased SA-*β*-gal activity compared with pLenti-mock control cells (Figure 1b, upper panels). Following CST1 knockdown, cell populations exhibiting SA-*β*-gal-positive staining increased to 70–80% and 55–90% in MDA-MB-231 and SW480 cells, respectively (Figure 1b, bottom graphs). Notably, the gene expression of representative SASP genes, including *interleukin-6* (*IL-6*) and *chemokine C-C motif ligand 20* (*CCL20*), was induced by CST1 knockdown in MDA-MB-231 and SW480 cells (Figure 1c). CST1 knockdown also significantly suppressed cell proliferation in MDA-MB-231 (Figure 1d) and SW480 (Supplementary Figure 2a) cells. Moreover, CST1 knockdown suppressed the colony growth of MDA-MB-231 (Figure 1e) and SW480 (Supplementary Figure 2b) cells under anchorage-independent conditions.

Based on these results, we examined cancer growth in a xenograft animal model. We injected MDA-MB-231 cells into the left (mock) and right (shCST1) flanks of 6-week-old female BALB/c athymic nude mice. Although MDA-MB-231-mock cells grew steadily, CST1 knockdown MDA-MB-231 cells exhibited significantly suppressed *in vivo* tumor growth (Figure 1f). The inhibition of tumor cell growth may result from G_0_/G_1_-phase cell cycle arrest (Supplementary Figures 3a and b) induced by a decrease in the expression of *cyclin D1* and *cyclin-dependent kinase 2* (*CDK2*) expression (Supplementary Figures 3c and d). To confirm that the G_0_/G_1_-phase cell cycle arrest was caused by CST1 knockdown, we conducted western blotting and found that CST1 knockdown suppressed cyclin D1 and phospho-retinoblastoma (p-Rb) and induced p21 (Figure 1g). These results suggest that CST1 knockdown reduces cancer cell proliferation through the induction of cellular senescence.

### Extracellular CST1 is involved in the prevention of cellular senescence

**Figure 2:**
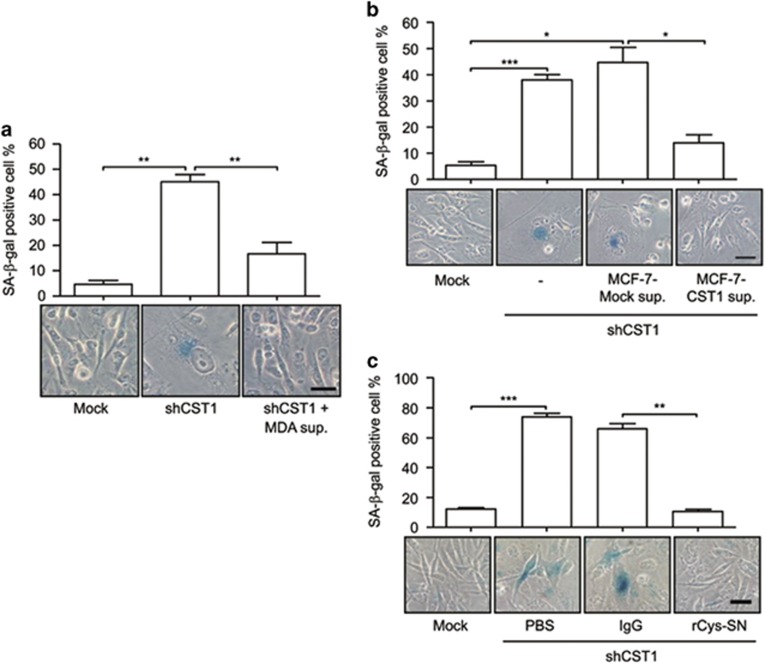
Extracellular CST1 is required in the prevention of cellular senescence. MDA-MB-231 cells transduced with a control shRNA (mock) or an shRNA for CST1 were cultured with an MDA-MB-231 culture supernatant (**a**) or CST1-overexpressing MCF-7 culture supernatant (**b**). (**c**) The effect of recombinant CST1-immunoglobulin (rCys-SN) on CST1 knockdown-mediated cellular senescence. Scale bars, 50 *μ*m. *P<0.05; **P<0.01; ***P<0.001 by Student's *t*-test

Although CST1 is a secretory protein, it was also present in the total cell lysates of MDA-MB-231 and SW480 cells (Supplementary Figure 1). To determine whether extracellular CST1 functions in the prevention of cellular senescence, we cultured shCST1-MDA-MB-231 cells with conditioned medium obtained from MDA-MB-231 cells. The supplementation of MDA-MB-231-conditioned medium suppressed both SA-*β*-gal activity and senescence-associated cell morphologies, including the enlarged nucleus and flattened cytoplasm (Figure 2a). To eliminate the possibility of unknown factors from MDA-MB-231 cells influencing the induction of cellular senescence, we established MCF-7 cells that stably expressed CST1 (Supplementary Figure 4a), because MCF-7 cells do not naturally express CST1 (Supplementary Figure 1). We found that SA-*β*-gal activity in shCST1-MDA-MB-231 cells was inhibited by supplementation with conditioned medium obtained from MCF-7 cells stably expressing CST1, but not by supplementation with wild-type MCF-7 conditioned medium (Figure 2b). To determine whether the increased SA-*β*-gal activity was solely induced by knockdown of CST1 in MDA-MB-231, MDA-MB-231 cells were seeded in a six-well plates coated with immunoglobulin G (IgG) or purified recombinant CST1-Ig (rCys-SN) (Supplementary Figure 4b). rCys-SN effectively suppressed SA-*β*-gal activity induced by CST1 knockdown compared with those treated with phosphate-buffered saline (PBS) or IgG alone (Figure 2c). These results show that extracellular CST1 is critical for the inhibition of cellular senescence.

### Downregulation of extracellular CatB activity by CST1 knockdown induces cellular senescence

**Figure 3:**
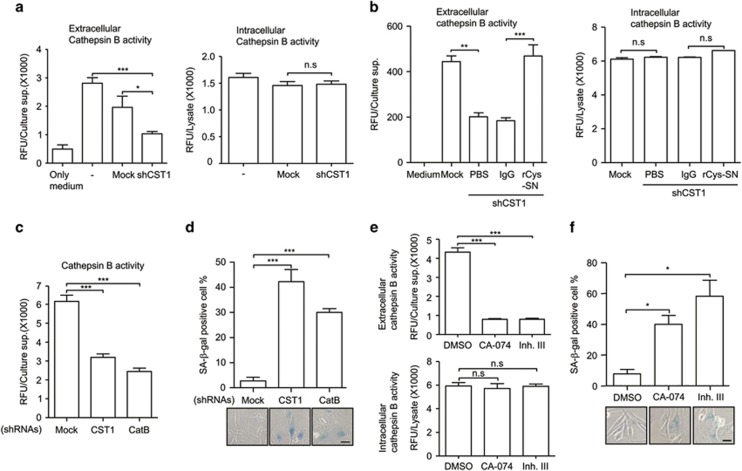
Downregulation of CatB activity by CST1 knockdown induces cellular senescence. (**a**) CatB activity was measured in the culture supernatant (left) or cell lysate (right) of CST1 knockdown cells. CatB activity of the culture supernatant (left) or cell lysate (right) from rCys-SN treated cells (**b**), or culture supernatant of CatB knockdown cells (**c**) was analyzed using a CatB activity fluorometric assay kit. (**d**) Effect of CatB knockdown on cellular senescence. After CST1 or CatB knockdown, cellular senescence was quantified by calculating the percentage of SA-*β*-gal-positive cells. To confirm the function of CatB associated with cellular senescence, MDA-MB-231 cells were treated with CatB inhibitors. The efficacy of the inhibitors was confirmed by the analysis of extracellular or intracellular CatB activity (**e**), and cellular senescence induced by the inhibitors was quantified by SA-*β*-gal staining (**f**). Scale bars, 50 *μ*m. Data were obtained from three independent experiments, and values are presented as the means±S.E.M. *P<0.05; **P<0.01; ***P<0.001 by Student's *t*-test

As CST1 is a known representative inhibitor of CatB that is located in the extracellular compartment and lysosomes, we examined the role of CatB on senescence induced by CST1 knockdown. Unexpectedly, we found that CST1 knockdown suppressed extracellular, but not intracellular, CatB activity (Figure 3a). To exclude the possibility that CST1 knockdown affects CatB expression levels, we investigated CatB expression levels in the extracellular or intracellular compartment (Supplementary Figure 5). CatB protein levels were unchanged under CST1 knockdown conditions. To examine the CST1-mediated positive regulation on CatB activity, we analyzed CatB activity in the culture supernatant obtained from CST1 knockdown MDA-MB-231 cells replenished with rCys-SN and observed that the reduced CatB activity caused by CST1 knockdown was restored with the addition of rCys-SN (Figure 3b). To confirm whether this reduced CatB activity was directly associated with CST1 knockdown-mediated cellular senescence, we inhibited CatB activity using a shRNA for CatB (Figure 3c), or chemical inhibitors of CatB (CA-074 or inhibitor III; Figure 3e). Notably, the SA-*β*-gal-positive cell population was significantly increased by CatB knockdown (Figure 3d) and CatB inhibitor treatment (Figure 3f).

Recently, it has been reported that CST1 neutralizes the inhibitory effect of cystatin C (CST3) on CatB activity.^28^ Therefore, we investigated whether cystatin C was associated with the regulatory function of CST1 on extracellular CatB activity. CST3 knockdown rescued extracellular CatB activity (Supplementary Figures 6a and b) and significantly inhibited SA-*β*-gal activity (Supplementary Figure 6c) in CST1 knockdown MDA-MB-231 cells. These results show that extracellular CatB activity is positively regulated by CST1 and is required for the prevention of cell senescence.

### CST1 knockdown induces cellular senescence through modulation of GSK3*β* activity

**Figure 4:**
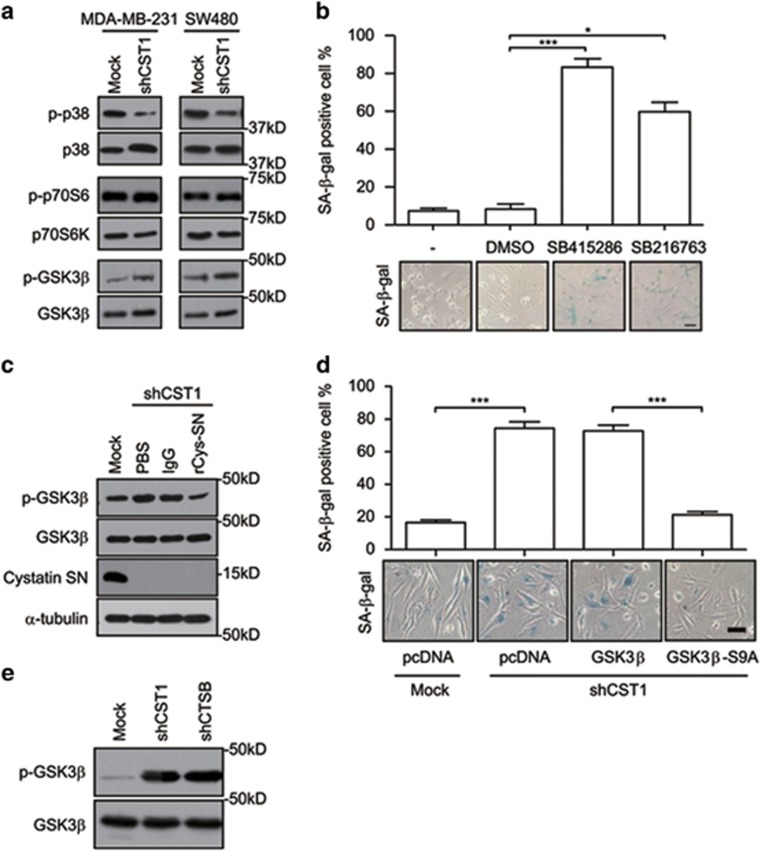
CST1 knockdown-mediated cellular senescence is mediated through GSK3*β* activity. (**a**) The phosphorylation of each kinase in CST1 knockdown cells was confirmed by western blotting. (**b**) The effect of GSK3*β* inhibitors on cellular senescence. MDA-MB-231 cells were cultured for 96 h in the presence of dimethyl sulfoxide or two different GSK3*β* inhibitors. Cellular senescence was quantified as the percentage of SA-*β-*gal-positive cells (upper) and photographed (lower). (**c**) Modulation of inhibitory phospho-GSK3*β* mediated by extracellular CST1. CST1 knockdown cells were cultured in the absence or presence of recombinant CST1-immunoglobulin (rCys-SN). (**d**) The inhibitory effect of active GSK3*β* on CST1 knockdown-mediated cellular senescence. MDA-MB-231 cells were pretransfected with pcDNA-HA, pcDNA-GSK3*β*-HA, and pcDNA-GSK3*β*-S9A-HA plasmids 24 h before the transduction of a control shRNA or an shRNA for CST1. (**e**) Modulation of phopho-GSK3*β* in CatB knockdown cells. Scale bars, 50 *μ*m. *P<0.05; ***P<0.001 by Student's *t*-test

To explore how CST1 inhibits cellular senescence, a human phospho-mitogen-activated protein kinase (MAPK) array containing p38MAPK, p70S6K and glycogen synthase kinase 3*β* (GSK3*β*) was used. Although the active phosphorylation of p70S6K (p-p70S6K) and inhibitory phosphorylation of GSK3*β* (p-GSK3*β*) were increased, the active phosphorylation of p38MAPK (p-p38) was decreased (Supplementary Figure 7a). The change in the phosphorylation status of GSK3*β* or p38MAPK was confirmed in both CST1 knockdown MDA-MB-231 and SW480 cancer cells (Figure 4a). Moreover, we found that the inhibition of GSK3*β* using GSK3*β* inhibitors, such as SB415286 and SB216763, induced SA-*β*-gal activity (Figure 4b). Conversely, a p38MAPK inhibitor, SB203580, did not induce SA-*β*-gal activity (Supplementary Figure 7b). These results indicate that the GSK3*β* signaling pathway is involved in CST1 knockdown-mediated cellular senescence. To examine whether GSK3*β* is modulated by extracellular CST1, we reconstituted CST1 knockdown MDA-MB-231 cells with rCys-SN. The increased GSK3*β* phosphorylation caused by CST1 knockdown was inhibited by the addition of rCys-SN (Figure 4c). To provide more evidence that GSK3*β* activity is directly involved in CST1 knockdown-mediated cellular senescence, we introduced wild-type or mutant GSK3*β* (GSK3*β*-S9A) expression vectors into MDA-MB-231 cells. Although SA-*β*-gal activity induced by CST1 knockdown was unaltered in mock vector and wild-type GSK3*β*-expressing cells, the ectopic expression of GSK3*β*-S9A (active form) significantly suppressed the SA-*β*-gal activity induced by CST1 knockdown (Figure 4d). CatB knockdown also induced the inhibitory phosphorylation of GSK3*β* at serine 9 (Figure 4e). These results show that CST1 knockdown induces cellular senescence through the inhibition of GSK3*β* activity, which is mediated by extracellular CatB activity.

### Inhibition of GSK3*β* activity by CST1 knockdown induces glycogen accumulation

**Figure 5:**
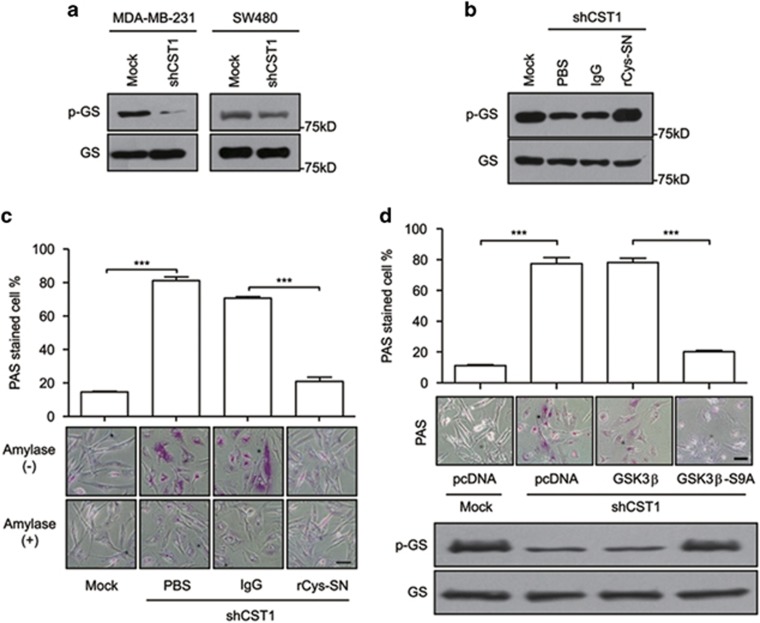
Downregulation of GSK3*β* activity mediated by CST1 knockdown induces cellular glycogen accumulation. (**a** and **b**) Modulation of phospho-GS in CST1 knockdown cells. MDA-MB-231 cells were transduced with a control short hairpin (shRNA) or shCST1 (**a**) and, in some experiments, cells were replenished with rCys-SN (**b**). (**c** and **d**) Analysis of glycogen accumulation in the cytosol. MDA-MB-231 cells were transduced with a control shRNA or shCST1 in the presence or absence of rCys-SN (**c**) and, in some experiments, transduction was performed after transfection with pcDNA, pcDNA-GSK3*β* or pcDNA-GSK3*β*-S9A (**d**) Glycogen accumulation was quantified as the percentage of PAS staining-positive cells (magenta) and photographed. Scale bars, 50 *μ*m. ***P<0.001 by Student's *t*-test

Reportedly, enhanced glycogenesis has been shown to be involved in cellular senescence via GSK3*β*/glycogen synthase (GS) modulation.^29^ Therefore, we investigated whether the inhibition of GSK3*β* induced the dephosphorylation of GS, thereby activating it and enhancing glycogenesis, which may induce cellular senescence. First, we confirmed that the dephosphorylation of GS was regulated by extracellular CST1. Knockdown of CST1 induced active GS (Figure 5a), whereas the reconstitution of CST1 knockdown cells with rCys-SN induced the phosphorylation of GS to its inactive form (Figure 5b), a process associated with glycogen accumulation (Figure 5c). As CST1 downregulated extracellular CatB activity, we confirmed that direct CatB inhibition, using shCatB or CA-074, decreased phosphorylation of GS in MDA-MB-231 cells (Supplementary Figure 8). To examine whether this glycogen accumulation was associated with the CST1-mediated modulation of GSK3*β* activity, we ectopically expressed wild-type GSK3*β* or GSK3*β*-S9A in CST1 knockdown MDA-MB-231 cells. Although glycogen accumulation was strongly observed in mock or wild-type GSK3*β*-expressing CST1 knockdown cells, the ectopic expression of GSK3*β*-S9A abrogated glycogen accumulation (Figure 5d). Following the direct inhibition of GSK3*β* using the chemical inhibitors, SB415286 or SB216763, glycogen accumulation was also observed (Supplementary Figure 9). These results imply that extracellular CST1-mediated CatB activity influences the GSK3*β*-GS pathway to regulate the glycogen metabolism associated with cellular senescence.

### Glycogen accumulation induced by GS is a direct cause of cellular senescence mediated by CST1 knockdown

**Figure 6:**
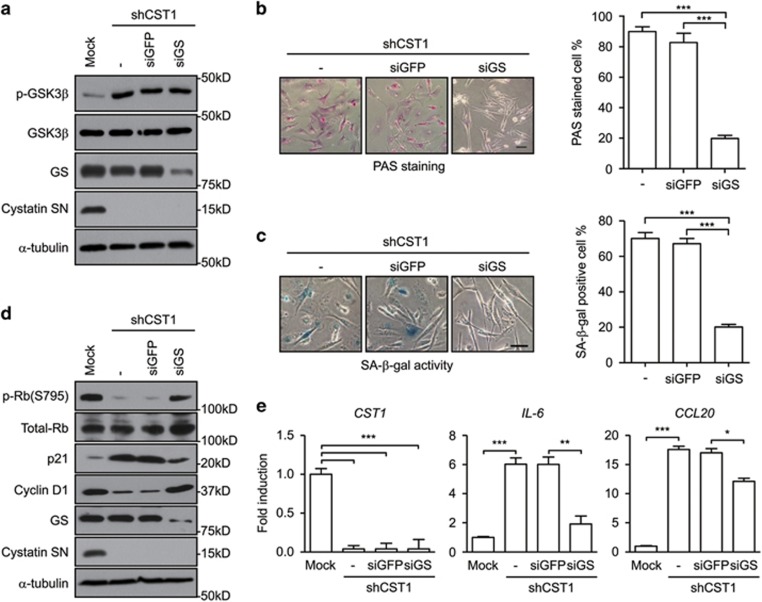
Cellular senescence is induced by GS activation resulted in glycogen accumulation in CST1 knockdown cells. The following assays were performed in CST1 knockdown MDA-MB-231 cells transfected with short-interfering RNAs (siRNAs) for green fluorescent protein (GFP) or GS. (**a** and **d**) The expression level of the indicated protein was analyzed by western blotting. (**b**) PAS staining analysis was performed to assess glycogen accumulation. (**c**) Quantification of cellular senescence was determined by the SA-*β*-gal assay. (**e**) Expression of SASP genes, *IL-6* and *CCL20*, was analyzed by real-time polymerase chain reaction. The results were normalized to the level of *β*-actin. Scale bars, 50 *μ*m. *P<0.05; **, *P*<0.01, *** *P*<0.001 by Student's *t*-test

To investigate the role of glycogen accumulation on cellular senescence in CST1 knockdown cells, we silenced GS in CST1 knockdown MDA-MB-231 cells. We confirmed that the knockdown of GS did not alter the phosphorylation of GSK3*β* at serine 9, which is upstream of GS (Figure 6a). Furthermore, we confirmed that GS knockdown suppressed glycogen accumulation in CST1 knockdown MDA-MB-231 cells (Figure 6b). Importantly, SA*-β*-gal-positive cells induced by CST1 knockdown were converted to SA-*β*-gal-negative cells following GS knockdown (Figure 6c). Associated with the inhibition of cell proliferation during cellular senescence, Rb phosphorylation and cyclin D1 expression, reduced by CST1 knockdown were recovered by GS knockdown in CST1 knockdown MDA-MB-231 cells. In addition, levels of the cell cycle regulator p21, which were increased by CST1 knockdown, were reduced by GS knockdown (Figure 6d). Similarly, the expression of SASP genes, including *IL-6* and *CCL20*, induced by CST1 knockdown was abrogated by GS knockdown (Figure 6e). These results show that extracellular CST1 negatively regulates GS, which induces abnormal glycogen accumulation, resulting in cellular senescence. Together, our results show that extracellular CST1 has an important role in tumorigenesis by preventing the cellular senescence induced by glycogen accumulation.

## Discussion

CST1 has been known as an endogenous inhibitor protein of cysteine proteases.^7^ CatB, a representative cysteine protease, is highly expressed in breast,^30^ colorectal^31^ and lung cancers.^32^ Moreover, CatB-mediated remodeling of the tumor environment directly affects cancer cell behaviors, such as invasion and metastasis.^33, 34, 35^ Paradoxically, CST1 has been proposed to be a novel marker in several tumors, including non-small cell lung cancer, pancreatic cancer, colorectal cancer and gastric cancer.^8, 9, 10, 11^ The upregulated expression of CST1 is associated with proliferation, invasion and metastasis of cells, which are the main characteristics of tumor malignancy.^8, 10, 36^ To date, the cooperative effect of CatB and CST1 during tumorigenesis remains poorly defined. In this study, we observed that extracellular CatB activity was positively regulated by CST1 expression and negatively regulated GS activity, inducing abnormal glycogen accumulation, which is a direct cause of cellular senescence.

Senescent cells are characterized by growth arrest, resistance to apoptosis, altered gene expression and the expression of senescence markers.^16^ Senescent cells usually exhibit G_1_-phase cell cycle arrest, which is caused by the expression of cell cycle inhibitors, such as p21 and p16.^21, 27, 37^ The most useful marker for identifying senescent cells is SA-*β*-gal.^23^ Furthermore, senescent cells are known to secrete SASP proteins, such as IL-6, IL-8 and CCL20, into the culture medium.^16, 38, 39^ In this study, senescence phenotypes, including growth arrest accompanying G_1_-phase cell cycle arrest, SA-*β*-gal activity and increased SASP secretion, were induced by the shRNA-mediated knockdown of CST1. *CST1* encodes a typical secretory protein presenting a secretory signal peptide at the N-terminus.^7^ When we treated CST1 knockdown cells with CST1-enriched conditioned medium or rCys-SN, CST1 knockdown-mediated cellular senescence was significantly inhibited. These results suggest that extracellular CST1 prevents cellular senescence. Recently, our colleagues reported that CST1 increases CatB activity by neutralizing the function of cystatin C,^28^ a known inhibitor of CatB with a broader spectrum of inhibitory activity than CST1.^40^ CST1 knockdown significantly decreased extracellular CatB activity without decreasing intracellular CatB activity. Expectedly, CatB knockdown also effectively induced cellular senescence. In addition, two independent inhibitors of CatB, CA-074 and cathepsin inhibitor III, induced cellular senescence. In particular, CA-074 is known to be non-permeable, potent, and specific a CatB inhibitor.^41, 42^ Our results suggested that extracellular CST1 contributes to the maintenance of extracellular CatB activity, thereby inhibiting cellular senescence. A recent study demonstrated that lysosomal CatB is released into the cytosol where it cleaves sirtuin 1, which mediates stress-induced premature senescence under stress conditions.^43^ Therefore, we propose that CatB has two opposing roles on cellular senescence depending on its localization. Although extracellular CatB prevents cellular senescence under normal conditions, intracellular lysosomal CatB induces cellular senescence under stress conditions.

We screened and confirmed signaling pathways involved in CST1 knockdown-mediated senescence using a human phospho-MAPK array and western blotting, and discovered that the inhibitory phosphorylation of GSK3*β* was regulated by extracellular CST1 and CatB. Unfortunately, we did not reveal how GSK3*β* phosphorylation was regulated under CST1 knockdown conditions. GSK3*β* is a multifunctional serine–threonine kinase that functions in various signaling pathways. It was first identified as an enzyme that regulates glycogen metabolism through GS inactivation.^44, 45^ GSK3*β* phosphorylation at the serine 9 residue creates an inactive form of GSK3*β*. This inactive form of GSK3*β* induces an increase in dephosphorylated GS, resulting in glycogen accumulation.^46^ Abnormal glycogen accumulation, which inhibits glucose utilization, has been shown to induce cellular senescence in various models.^29, 47, 48^ The abnormal glycogen accumulation induced by CST1 or CatB knockdown may trigger intracellular signal transduction because glycogen directly activates adenosine monophosphate-activated protein kinase,^49^ which can lead to cellular senescence if persistently activated.^50^ In this study, the inhibitory phosphorylation of GSK3*β* was increased under CST1 or CatB knockdown, resulting in glycogen accumulation and cellular senescence. Conversely, a constitutively active mutant GSK3*β* (GSK3*β*-S9A) inhibited the glycogen accumulation and cellular senescence induced by CST1 knockdown. CST1 or CatB knockdown also induced a decrease in GS phosphorylation and glycogen accumulation, which were effectively inhibited by GSK3*β*-S9A. GS knockdown significantly diminished cellular senescence phenotypes, including SA-*β*-gal activity, cell cycle regulator modulation and SASP gene expression, as well as inhibiting glycogen accumulation in CST1 knockdown cells.

To date, studies on extracellular CatB and CST1 have focused on cell proliferation, invasion and metastasis related to extracellular matrix degradation during tumorigenesis. In this study, we revealed a new function of the extracellular CST1–CatB axis in the modulation of glycogen metabolism via the regulation of the GSK3*β*–GS axis, contributing to the prevention of cellular senescence. Finally, we suggest that extracellular CST1, a positive regulator of extracellular CatB, contributes to tumorigenesis by preventing cellular senescence through the inhibition of abnormal glycogen accumulation.

## Materials and methods

### Cell culture and reagents

MCF-7, MDA-MB-231 and HEK 293T/17 cells (American Type Culture Collection, Manassas, VA, USA), and Colo205, Lovo, SW480, SW620, DLD-1, HCT116, HT29, LS174T and SNUC1 cells (Korean Cell Line Bank, Seoul, Republic of Korea) were cultured in Dulbecco's modified Eagle's medium (DMEM; Sigma-Aldrich Corp., St. Louis, MO, USA) supplemented with 10% (v/v) heat-inactivated fetal bovine serum (Sigma-Aldrich Corp.) and 1% (v/v) penicillin/streptomycin solution (Lonza, Basel, Switzerland) at 37 °C in a 5% CO_2_ incubator. In some experiments, cells were treated with specific inhibitors (Calbiochem, San Diego, CA, USA) of GSK3*β* (SB415286 or SB216763), p38MAPK (SB203580) or CatB (CA-074 or cathepsin inhibitor III).

### Western blot and phopho-MAPK array

Cell lysates were extracted with CETi protein extraction solution (TransLab, Daejeon, Korea). Thirty to fifty micrograms of protein extract was used for western blotting with antibodies against p-Rbs, p21, cyclin D1, GSK3*β*, p-GSK3*β*, CatB, GS, p-GS, p38MAPK, p-p38MAPK, p70S6K, p-p70S6K (Cell Signaling, Denvers, MA, USA), *α*-tubulin, mouse IgG peroxidase conjugate, rabbit IgG peroxidase conjugate (Sigma-Aldrich Corp.) and Rb (Santa Cruz Biotechnology, Inc., Dallas, TX, USA). The human phospho-MAPK Array Kit (R&D Systems, Inc., Minneapolis, MN, USA) was used according to the manufacturer's instructions. A polyclonal antibody against CST1 was obtained from mice immunized with the purified protein.

### Cell transfection and RNA interference

pcDNA-HA, pcDNA-CST1-HA, pcDNA-GSK3*β*-HA and pcDNA-GSK3*β*S9A-HA plasmids were transfected into cells using FuGENE HD Transfection Agent (Promega Corp., Madison, WI, USA). For *CST1*, *CST3* and *CatB* knockdown, validated shRNAs against the genes were purchased from Sigma-Aldrich Corp., and viral production and infection of cell lines were performed according to the ViraPower Lentiviral Expression System protocol (Invitrogen, Carlsbad, CA, USA). The sequence of the shRNA constructs were as follows: shCST1-1, 5′-CCGGGAAGAAACAGTTGTGCTCTTTCTCGAGAAAGAGCACAACTGTTTCTTCTTTTT-3′ shCST1–2, 5′-CCGGCCAGGCCATTCGCACCAGCCACTCGAGTGGCTGGTGCGAATGGCCTGGTTTTT-3′ shCST3, 5′-CCGGCCAGGCCATTCGCACCAGCCACTCGAGTGGCTGGTGCGAATGGCCTGGTTTTT-3′ shCatB, 5′-CCGGCTGTGCCTATCACCTCTTATGCTCGAGCATAAGAGGTGATAGGCACAGTTTTTG-3′. siRNAs targeting GS (siGS; 5′-GAAUCCUUAUCCAGGCUAA-3′) and control siRNA (siGFP; 5′-GUUCAGCGUGUCCGGCGAGTT-3′) were purchased from Genolution Inc. (Seoul, Republic of Korea) and transfected into cells using Lipofectamine 3000 (Invitrogen).

### SA-*β*-gal staining

SA-*β*-gal activity in cells was determined by cytochemical staining. Senescent cells were quantified as the number of SA-*β*-gal-positive cells per total cell counts in the same microscopic field. The number was determined by counting at least five random fields per sample.

### Cell proliferation assay

For the analysis of anchorage-dependent cell proliferation, cells were seeded on to six-well plates at a density of 5 × 10^4^ cells per well, and cell numbers were counted daily for 6 days. Anchorage-independent cell proliferation was determined by the colony forming assay. Cells (1 × 10^5^ cells per well) were suspended in complete DMEM containing 0.35% agarose and laid over agar consisting of complete DMEM containing 0.5% agarose on a 12-well plate. Cells were cultured for 12 days, and colonies that developed were stained with 0.005% crystal violet and counted using light microscopy. For the analysis of xenograft tumor growth, cells (1 × 10^6^ cells) mixed with Matrigel (BD Biosciences, San Jose, CA, USA) were subcutaneously injected into the right flank for shCST1 or left flank for the control shRNA of 6-week-old female athymic nude mice (Orient Bio Co., Seongnam, Republic of Korea). Tumor volume was calculated using the following equation: (maximal length × perpendicular width × height)/2.

All procedures were approved by the Animal Ethical Committee of Gyeongsang National University, Jinju, Republic of Korea.

### Reverse transcription and real-time polymerase chain reaction

Total RNA was extracted from cells using RiboEx (GeneAll Biotechnology Co., Ltd., Seoul, Republic of Korea), and cDNA was synthesized using a reverse transcription kit (Thermo Fisher Scientific, Inc., Waltham, MA, USA). Reverse transcription polymerase chain reaction (PCR) was performed using the DNA Engine Dyad Peltier Thermal Cycler (Bio-Rad Laboratories, Inc., Hercules, CA, USA). Real-time PCR was performed using SSoFast EvaGreen Supermix and a CFX96 Real-Time PCR Detection System (Bio-Rad Laboratories, Inc.). The following primers were used for amplification: *CST1*, 5′-CCATGGCCCAGTATCTGAGT-3′ and 5′-GAAGGCACAGGTGTCCAAGT-3′ *CST3*, 5′-GCAAGCCGCCGCGCCTG-3′ and 5′-ATTGTGCCCTGCCAAGGC-3′ *IL-6*, 5′-TACCCCCAGGAGAAGATTCC-3′ and 5′-TTTTCTGCCAGTGCCTCTTT-3′ *CCL20*, 5′-GCAAGCAACTTTGACTGCTG-3′ and 5′-TCACCCAAGTCTGTTTTGGA-3′ *cyclin D1,* 5′-CCTAAGTTCGGTTCCGATGA-3′ and 5′-ACGTCAGCCTCCACACTCTT-3′ *CDK2,* 5′-CATTCCTCTTCCCCTCATCA-3′ and 5′-CAGGGACTCCAAAAGCTCTG-3′ *GAPDH*, 5′-CCATCACCATCTTCCAGGAG-3′ and 5′-ACAGTCTTCTGGGTGGCAGT-3′ *β-actin*, 5′-GGTCATCACTATTGGCAACG-3′ and 5′-ACGGATGTCAACGTCACACT-3′.

### Determination of cathepsin activity

Cells were cultured in complete protein-free medium (Lonza). Cathepsin activity in the cell culture supernatant was quantified using a fluorometric assay kit (BIoVision Inc., Milpitas, CA, USA) according to the manufacturer's recommendations.

### PAS staining assay

Glycogen staining was performed using the periodic acid–Schiff (PAS) reagent (Sigma-Aldrich Corp.). Cells were fixed in formalin–ethanol solution for 1 min at room temperature and stained with 0.5% Schiff's reagent for 15 min. After washing cells with PBS several times, stained cells were observed and photographed. Cells accumulating glycogen were quantified as the number of PAS-positive cells (magenta) per total cell count in the same microscopic field. The number was determined by counting at least five random fields per sample. To determine the origin of the staining, cells were treated with 0.5% amylase (Sigma-Aldrich Corp.) for 90 min at 37 °C to degrade intracellular glycogen before PAS staining.

### Statistical analysis

Data related to cell proliferation were analyzed using analysis of variance (ANOVA), and all other results were analyzed using the unpaired Student's *t*-test. A *P*-value of <0.05 was considered statistically significant.

## References

[bib1] Turk V, Turk B, Guncar G, Turk D, Kos J. Lysosomal cathepsins: structure, role in antigen processing and presentation, and cancer. Adv Enzyme Regul 2002; 42: 285–303. 12123721 10.1016/s0065-2571(01)00034-6

[bib2] Buck MR, Karustis DG, Day NA, Honn KV, Sloane BF. Degradation of extracellular-matrix proteins by human cathepsin B from normal and tumour tissues. Biochem J 1992; 282: 273–278. 1540143 10.1042/bj2820273 PMC1130919

[bib3] Premzl A, Zavasnik-Bergant V, Turk V, Kos J. Intracellular and extracellular cathepsin B facilitate invasion of MCF-10 A neoT cells through reconstituted extracellular matrix *in vitro*. Exp Cell Res 2003; 283: 206–214. 12581740 10.1016/s0014-4827(02)00055-1

[bib4] Roshy S, Sloane BF, Moin K. Pericellular cathepsin B and malignant progression. Cancer Metastasis Rev 2003; 22: 271–286. 12785001 10.1023/a:1023007717757

[bib5] Bao W, Fan Q, Luo X, Cheng WW, Wang YD, Li ZN et al. Silencing of cathepsin B suppresses the proliferation and invasion of endometrial cancer. Oncol Rep 2013; 30: 723–730. 23708264 10.3892/or.2013.2496

[bib6] Bian B, Mongrain S, Cagnol S, Langlois MJ, Boulanger J, Bernatchez G et al. Cathepsin B promotes colorectal tumorigenesis, cell invasion, and metastasis. Mol Carcinog 2016; 55: 671–687. 25808857 10.1002/mc.22312 PMC4832390

[bib7] Dickinson DP. Salivary (SD-type) cystatins: over one billion years in the making—but to what purpose? Crit Rev Oral Biol Med 2002; 13: 485–508. 12499242 10.1177/154411130201300606

[bib8] Cao X, Li Y, Luo RZ, Zhang L, Zhang SL, Zeng J et al. Expression of cystatin SN significantly correlates with recurrence, metastasis, and survival duration in surgically resected non-small cell lung cancer patients. Sci Rep 2015; 5: 8230. 25648368 10.1038/srep08230 PMC4316172

[bib9] Jiang J, Liu HL, Liu ZH, Tan SW, Wu B. Identification of cystatin SN as a novel biomarker for pancreatic cancer. Tumour Biol 2015; 36: 3903–3910. 25577248 10.1007/s13277-014-3033-3

[bib10] Choi EH, Kim JT, Kim JH, Kim SY, Song EY, Kim JW et al. Upregulation of the cysteine protease inhibitor, cystatin SN, contributes to cell proliferation and cathepsin inhibition in gastric cancer. Clin Chim Acta 2009; 406: 45–51. 19463800 10.1016/j.cca.2009.05.008

[bib11] Yoneda K, Iida H, Endo H, Hosono K, Akiyama T, Takahashi H et al. Identification of cystatin SN as a novel tumor marker for colorectal cancer. Int J Oncol 2009; 35: 33–40. 19513549

[bib12] Hayflick L. The limited *in vitro* lifetime of human diploid cell strains. Exp Cell Res 1965; 37: 614–636. 14315085 10.1016/0014-4827(65)90211-9

[bib13] d'Adda di Fagagna F. Living on a break: cellular senescence as a DNA-damage response. Nat Rev Cancer 2008; 8: 512–522. 18574463 10.1038/nrc2440

[bib14] Harley CB, Futcher AB, Greider CW. Telomeres shorten during ageing of human fibroblasts. Nature 1990; 345: 458–460. 2342578 10.1038/345458a0

[bib15] Kuilman T, Michaloglou C, Mooi WJ, Peeper DS. The essence of senescence. Genes Dev 2010; 24: 2463–2479. 21078816 10.1101/gad.1971610 PMC2975923

[bib16] Campisi J, d'Adda di Fagagna F. Cellular senescence: when bad things happen to good cells. Nat Rev Mol Cell Biol 2007; 8: 729–740. 17667954 10.1038/nrm2233

[bib17] Ewald JA, Desotelle JA, Wilding G, Jarrard DF. Therapy-induced senescence in cancer. J Nat Cancer Ins 2010; 102: 1536–1546. 10.1093/jnci/djq364 PMC2957429 20858887

[bib18] Nardella C, Clohessy JG, Alimonti A, Pandolfi PP. Pro-senescence therapy for cancer treatment. Nat Rev Cancer 2011; 11: 503–511. 21701512 10.1038/nrc3057

[bib19] Zhu J, Woods D, McMahon M, Bishop JM. Senescence of human fibroblasts induced by oncogenic Raf. Genes Dev 1998; 12: 2997–3007. 9765202 10.1101/gad.12.19.2997 PMC317194

[bib20] Beausejour CM, Krtolica A, Galimi F, Narita M, Lowe SW, Yaswen P et al. Reversal of human cellular senescence: roles of the p53 and p16 pathways. EMBO J 2003; 22: 4212–4222. 12912919 10.1093/emboj/cdg417 PMC175806

[bib21] Serrano M, Lin AW, McCurrach ME, Beach D, Lowe SW. Oncogenic ras provokes premature cell senescence associated with accumulation of p53 and p16INK4a. Cell 1997; 88: 593–602. 9054499 10.1016/s0092-8674(00)81902-9

[bib22] Parrinello S, Samper E, Krtolica A, Goldstein J, Melov S, Campisi J. Oxygen sensitivity severely limits the replicative lifespan of murine fibroblasts. Nat Cell Biol 2003; 5: 741–747. 12855956 10.1038/ncb1024 PMC4940195

[bib23] Dimri GP, Lee X, Basile G, Acosta M, Scott G, Roskelley C et al. A biomarker that identifies senescent human cells in culture and in aging skin *in vivo*. Proc Natl Acad Sci USA 1995; 92: 9363–9367. 7568133 10.1073/pnas.92.20.9363 PMC40985

[bib24] Coppe JP, Patil CK, Rodier F, Sun Y, Munoz DP, Goldstein J et al. Senescence-associated secretory phenotypes reveal cell-nonautonomous functions of oncogenic RAS and the p53 tumor suppressor. PLoS Biol 2008; 6: 2853–2868. 19053174 10.1371/journal.pbio.0060301 PMC2592359

[bib25] Hanahan D, Weinberg RA. The hallmarks of cancer. Cell 2000; 100: 57–70. 10647931 10.1016/s0092-8674(00)81683-9

[bib26] Dimri GP. What has senescence got to do with cancer? Cancer cell 2005; 7: 505–512. 15950900 10.1016/j.ccr.2005.05.025 PMC1769521

[bib27] Campisi J. Cellular senescence as a tumor-suppressor mechanism. Trends Cell Biol 2001; 11: S27–S31. 11684439 10.1016/s0962-8924(01)02151-1

[bib28] Kim JT, Lee SJ, Kang MA, Park JE, Kim BY, Yoon DY et al. Cystatin SN neutralizes the inhibitory effect of cystatin C on cathepsin B activity. Cell Death Dis 2013; 4: e974. 24357805 10.1038/cddis.2013.485 PMC3877556

[bib29] Seo YH, Jung HJ, Shin HT, Kim YM, Yim H, Chung HY et al. Enhanced glycogenesis is involved in cellular senescence via GSK3/GS modulation. Aging Cell 2008; 7: 894–907. 18782348 10.1111/j.1474-9726.2008.00436.x

[bib30] Foekens JA, Kos J, Peters HA, Krasovec M, Look MP, Cimerman N et al. Prognostic significance of cathepsins B and L in primary human breast cancer. J Clin Oncol 1998; 16: 1013–1021. 9508185 10.1200/JCO.1998.16.3.1013

[bib31] Adenis A, Huet G, Zerimech F, Hecquet B, Balduyck M, Peyrat JP et al. L, and D activities in colorectal carcinomas: relationship with clinico-pathological parameters. Cancer Lett 1995; 96: 267–275. 7585467 10.1016/0304-3835(95)03930-u

[bib32] Sukoh N, Abe S, Ogura S, Isobe H, Takekawa H, Inoue K et al. Immunohistochemical study of cathepsin B. Prognostic significance in human lung cancer. Cancer 1994; 74: 46–51. 8004582 10.1002/1097-0142(19940701)74:1<46::aid-cncr2820740109>3.0.co;2-g

[bib33] Gondi CS, Rao JS. Cathepsin B as a cancer target. Expert Opin Ther Targets 2013; 17: 281–291. 23293836 10.1517/14728222.2013.740461 PMC3587140

[bib34] Khan A, Krishna M, Baker SP, Banner BF. Cathepsin B and tumor-associated laminin expression in the progression of colorectal adenoma to carcinoma. Mod Pathol 1998; 11: 704–708. 9720496

[bib35] Victor BC, Anbalagan A, Mohamed MM, Sloane BF, Cavallo-Medved D. Inhibition of cathepsin B activity attenuates extracellular matrix degradation and inflammatory breast cancer invasion. Breast Cancer Res 2011; 13: R115. 22093547 10.1186/bcr3058 PMC3326557

[bib36] Blanco MA, LeRoy G, Khan Z, Aleckovic M, Zee BM, Garcia BA et al. Global secretome analysis identifies novel mediators of bone metastasis. Cell Res 2012; 22: 1339–1355. 22688892 10.1038/cr.2012.89 PMC3434351

[bib37] Herbig U, Jobling WA, Chen BP, Chen DJ, Sedivy JM. Telomere shortening triggers senescence of human cells through a pathway involving ATM, p53, and p21(CIP1), but not p16(INK4a). Mol Cell 2004; 14: 501–513. 15149599 10.1016/s1097-2765(04)00256-4

[bib38] Young AR, Narita M. SASP reflects senescence. EMBO Rep 2009; 10: 228–230. 19218920 10.1038/embor.2009.22 PMC2658552

[bib39] Kuilman T, Peeper DS. Senescence-messaging secretome: SMS-ing cellular stress. Nat Rev Cancer 2009; 9: 81–94. 19132009 10.1038/nrc2560

[bib40] Abrahamson M, Dalboge H, Olafsson I, Carlsen S, Grubb A. Efficient production of native, biologically active human cystatin C by *Escherichia coli*. FEBS Lett 1988; 236: 14–18. 3042461 10.1016/0014-5793(88)80276-x

[bib41] Murata M, Miyashita S, Yokoo C, Tamai M, Hanada K, Hatayama K et al. Novel epoxysuccinyl peptides. Selective inhibitors of cathepsin B, *in vitro*. FEBS Lett 1991; 280: 307–310. 2013328 10.1016/0014-5793(91)80318-w

[bib42] Buttle DJ, Murata M, Knight CG, Barrett AJ. CA074 methyl ester: a proinhibitor for intracellular cathepsin B. Arch Biochem Biophys 1992; 299: 377–380. 1444478 10.1016/0003-9861(92)90290-d

[bib43] Chen J, Xavier S, Moskowitz-Kassai E, Chen R, Lu CY, Sanduski K et al. Cathepsin cleavage of sirtuin 1 in endothelial progenitor cells mediates stress-induced premature senescence. Am J Pathol 2012; 180: 973–983. 22234173 10.1016/j.ajpath.2011.11.033 PMC3349894

[bib44] Embi N, Rylatt DB, Cohen P. Glycogen synthase kinase-3 from rabbit skeletal muscle. Separation from cyclic-AMP-dependent protein kinase and phosphorylase kinase. Eur J Biochem 1980; 107: 519–527. 6249596

[bib45] Woodgett JR. Molecular cloning and expression of glycogen synthase kinase-3/factor A. EMBO J 1990; 9: 2431–2438. 2164470 10.1002/j.1460-2075.1990.tb07419.x PMC552268

[bib46] Cohen P, Frame S. The renaissance of GSK3. Nat Rev Mol Cell Biol 2001; 2: 769–776. 11584304 10.1038/35096075

[bib47] Favaro E, Bensaad K, Chong MG, Tennant DA, Ferguson DJ, Snell C et al. Glucose utilization via glycogen phosphorylase sustains proliferation and prevents premature senescence in cancer cells. Cell Metab 2012; 16: 751–764. 23177934 10.1016/j.cmet.2012.10.017

[bib48] Sinadinos C, Valles-Ortega J, Boulan L, Solsona E, Tevy MF, Marquez M et al. Neuronal glycogen synthesis contributes to physiological aging. Aging Cell 2014; 13: 935–945. 25059425 10.1111/acel.12254 PMC4331761

[bib49] McBride A, Ghilagaber S, Nikolaev A, Hardie DG. The glycogen-binding domain on the AMPK beta subunit allows the kinase to act as a glycogen sensor. Cell Metab 2009; 9: 23–34. 19117544 10.1016/j.cmet.2008.11.008 PMC2642990

[bib50] Jones RG, Plas DR, Kubek S, Buzzai M, Mu J, Xu Y et al. AMP-activated protein kinase induces a p53-dependent metabolic checkpoint. Mol Cell 2005; 18: 283–293. 15866171 10.1016/j.molcel.2005.03.027

