# Supplementary material for: Extracellular cystatin SN and cathepsin B prevent cellular senescence by inhibiting abnormal glycogen accumulation
Source: Cell Death Dis. 2017 Apr 6;8(4):e2729–. doi: 10.1038/cddis.2017.153 (PMC5477579; doi:10.1038/cddis.2017.153)
Supplement: Supplementary Information [file cddis2017153x1.docx]

**Supplementary Figure** **Legends**

**Supplementary Figure 1.** Expression profiles of *CST1* and cystatin SN in human colon and breast cancer cell lines. RT-PCR was performed to determine *CST1* transcript levels in the cell lines. The presence of CST1 in the culture supernatant or cell lysate was analyzed by western blotting.

**Supplementary Figure 2.** Effects of CST1 knockdown on SW480 cell proliferation.

(a) Analysis of anchorage-dependent cell proliferation. SW480 cells transduced by each shRNA were seeded on to 6-well plates (5 × 10^4^ cells/well) and cultured for the indicated number of days. The number of cells was counted using a hemocytometer. One-way ANOVA was used for statistical analysis (*, P < 0.05). (b) Analysis of anchorage-independent cell proliferation. Cells were seeded in complete DMEM containing agarose (1 × 10^5^ cells/well) and cultured for 12 days. Colonies were stained with 0.005% crystal violet and quantified using light microscopy. Scale bars, 10 μm. *, *P* < 0.05 by Student’s *t*-test.

**Supplementary Figure 3.** Cell-cycle arrest and related gene expression following CST1 knockdown.

(a and b) Cell-cycle analysis. MDA-MB-231 cells were transduced with the indicated shRNAs. Four days after transduction, the cells were fixed with 70% ethanol and stained with propidium iodide. Data were acquired by flow cytometry, and the cell cycle was analyzed using ModFit LT software. Transcript levels of *cyclin D1*, *CDK2*, and *CST1* in MDA-MB-231 cells (c) and SW480 cells (d) were determined by real-time PCR. The relative expression level of each target gene was normalized to that of *β-actin*. **, *P* < 0.01 by Student’s *t*-test.

**Supplementary Figure 4.** Determination of CST1 expression in the culture supernatant of CST1*-*overexpressing MCF-7 cells (a) and purified rCys-SN (b).

**Supplementary Figure 5.** CST1-knockdown does not affect CatB expression levels in MDA-MB-231 cells. MDA-MB-231 cells were transduced with control shRNA or shCST1, then the expression level of CatB in the cell lysate (*left*) or culture supernatant (*right*) was determined by western blotting. Coomassie blue staining confirmed equal protein loading.

**Supplementary Figure 6.** CST3 (cystatin C) knockdown rescues extracellular CatB activity and inhibits cellular senescence induced by CST1 knockdown. (a) The efficiency of CST1 or CST3 knockdown was confirmed by reverse transcription PCR. (b) The CatB activities in the indicated experimental groups were measured and compared. (c) The effect of CST3 knockdown on CST1 knockdown-mediated cellular senescence. **, *P* < 0.01; ***, *P* < 0.001 by Student’s *t*-test.

**Supplementary Figure 7.** Analysis of human phospho-MAPKs in CST1-knockdown MDA-MB-231 cells. (a) The relevant signals are highlighted by black frames; 1: p38MAPK, 2: p70S6 kinase, 3: GSK3β. (b) MDA-MB-231 cells were cultured for 4 days in the presence or absence of the p38 MAPK inhibitor, SB203580. The efficiency of p38 MAPK inhibition was confirmed by western blotting (*left*) and cellular senescence was determined by SA-β-gal assay (*right*).

**Supplementary Figure 8.** Down-regulation of CatB leads to the activation of glycogen synthase in MDA-MB-231 cells. Western blot analysis of glycogen synthase phosphorylation in CatB-knockdown (a) or CatB inhibitor (CA-074) treated cells (b).

**Supplementary Figure 9**. GSK3β inhibitors-mediated glycogen accumulation. MDA-MB-231 cells were treated with DMSO or one of two different GSK3β inhibitors, SB415286 or SB216763, and cultured for 4 days. GS phosphorylation was detected by western blotting (*bottom*) and glycogen accumulation was quantified as the percentage of PAS staining-positive cells (magenta; *upper*) and photographed (*middle*). Scale bars, 50 μm. **, *P* < 0.01; ***, *P* < 0.001 by Student’s *t*-test.
