# Supplementary figures and images for: Extracellular cystatin SN and cathepsin B prevent cellular senescence by inhibiting abnormal glycogen accumulation
Source: Cell Death Dis. 2017 Apr 6;8(4):e2729–. doi: 10.1038/cddis.2017.153 (PMC5477579; doi:10.1038/cddis.2017.153)

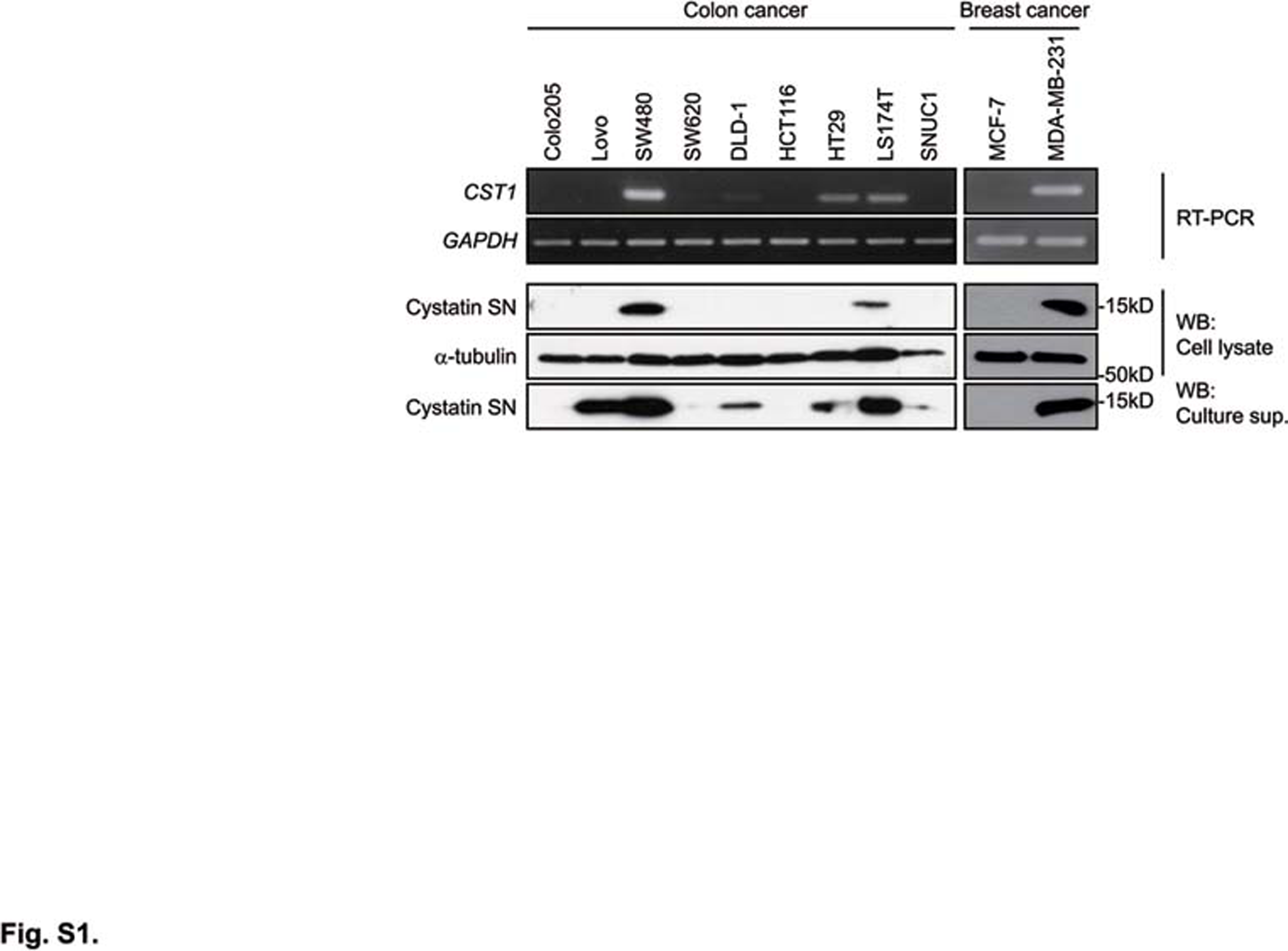

Supplement: Supplementary Figure 1 [file cddis2017153x2.tif]

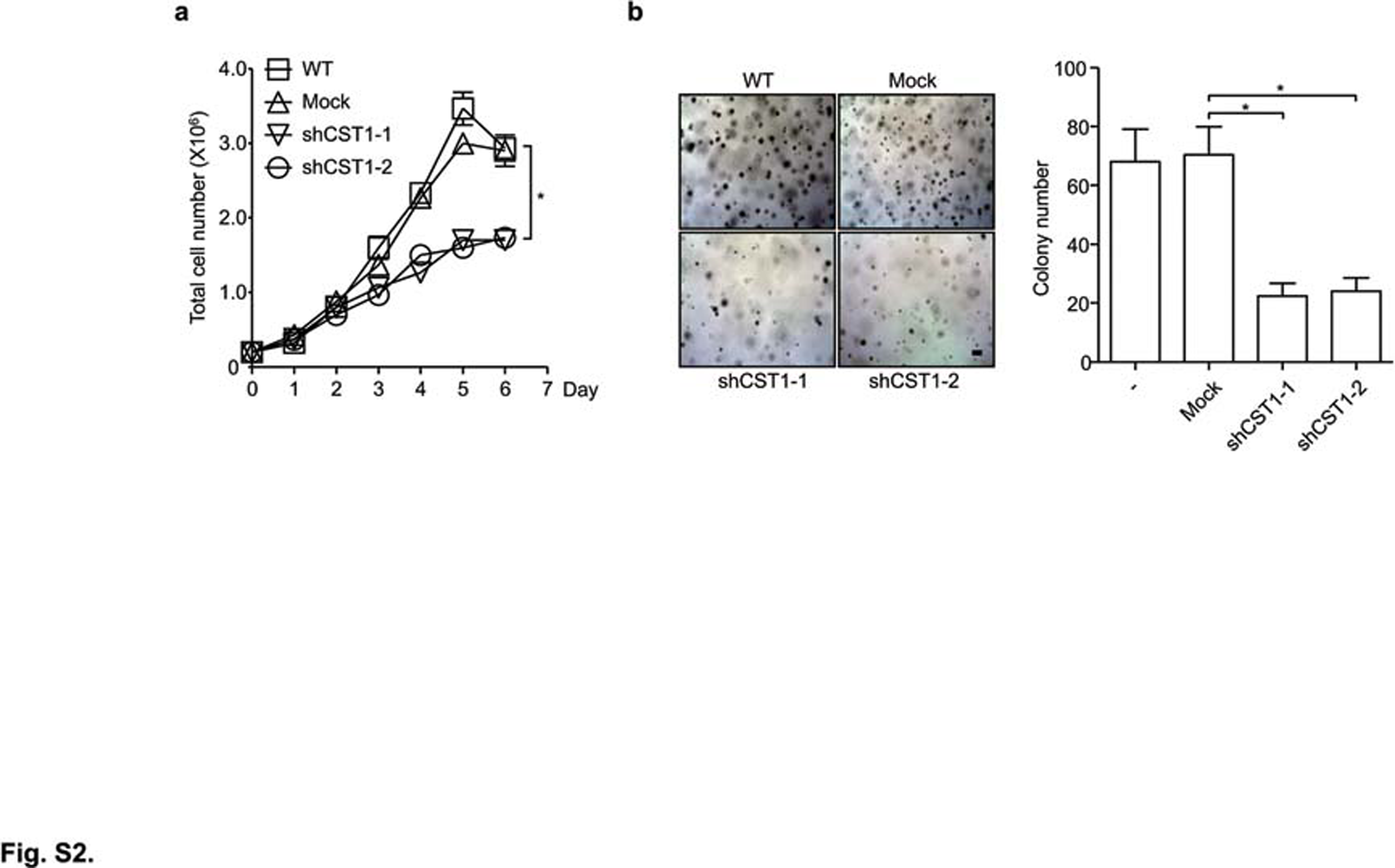

Supplement: Supplementary Figure 2 [file cddis2017153x3.tif]

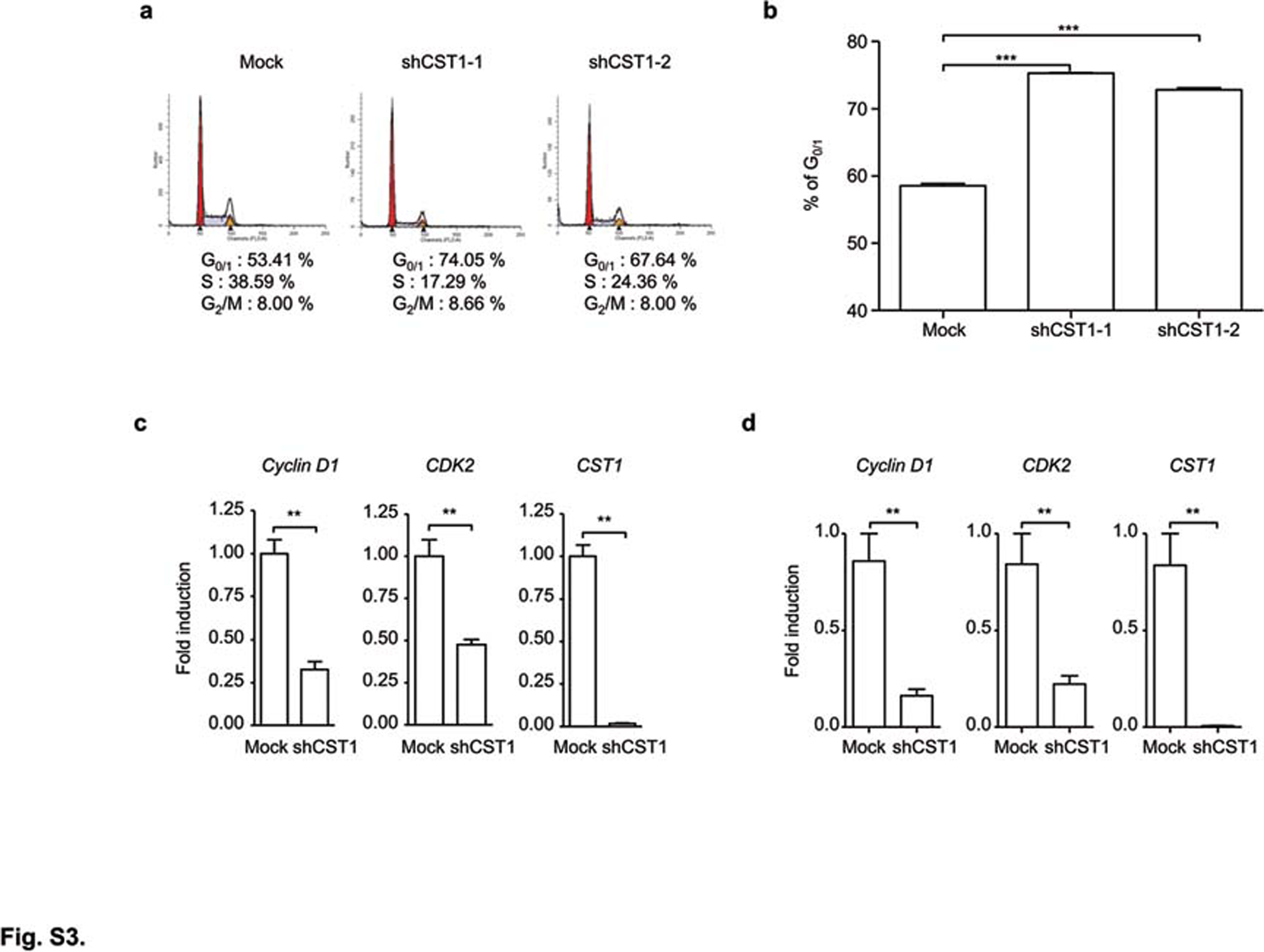

Supplement: Supplementary Figure 3 [file cddis2017153x4.tif]

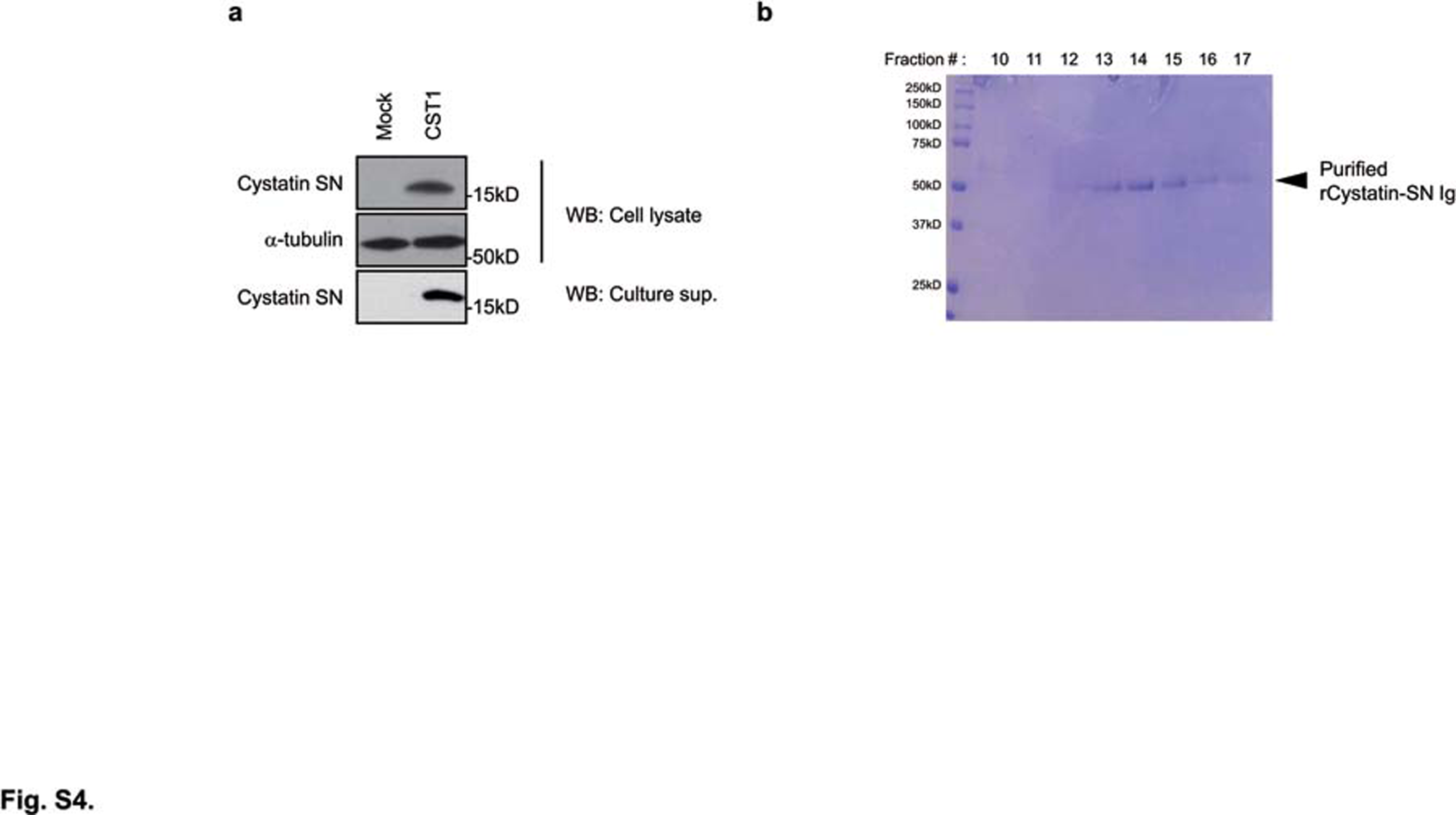

Supplement: Supplementary Figure 4 [file cddis2017153x5.tif]

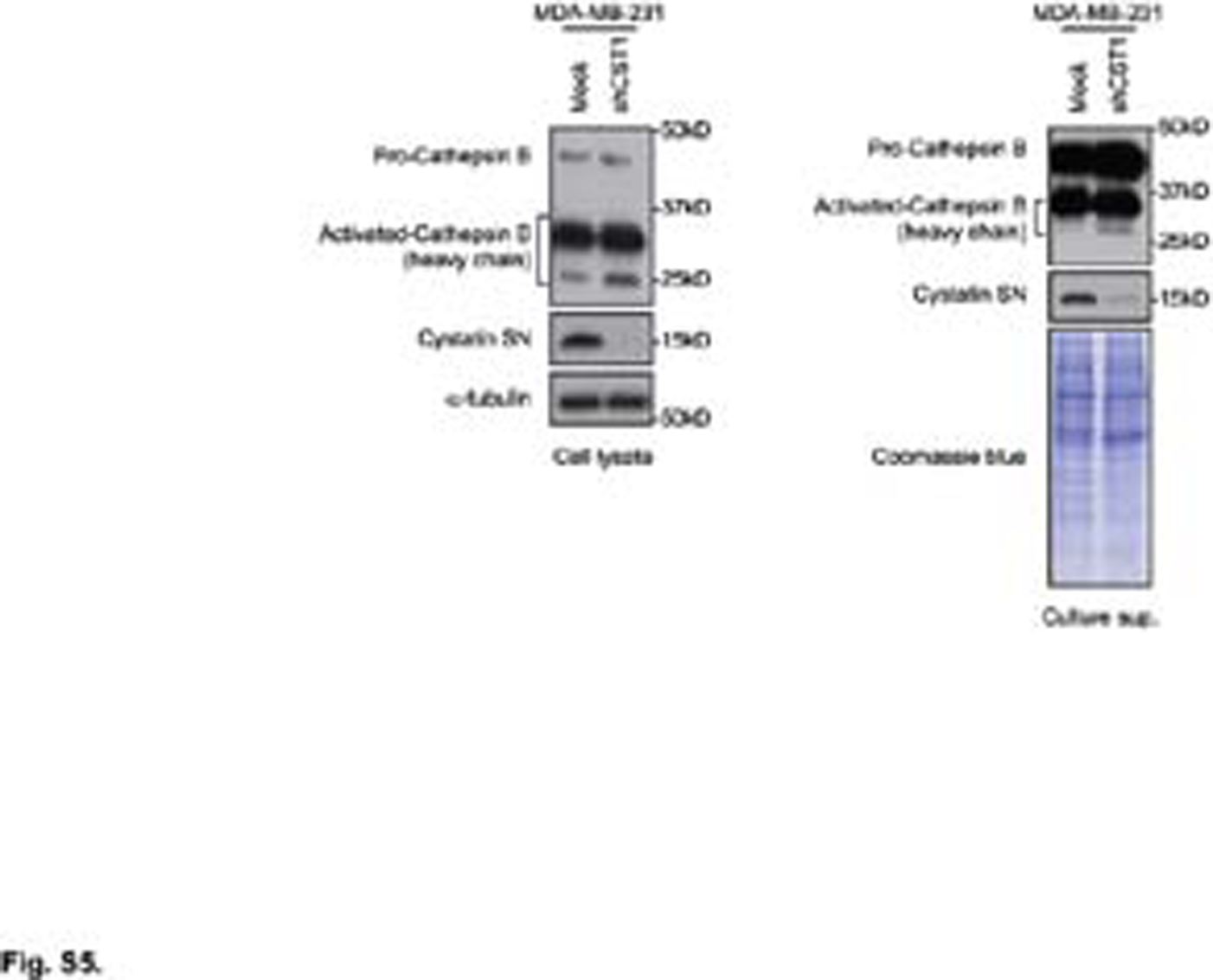

Supplement: Supplementary Figure 5 [file cddis2017153x6.tif]

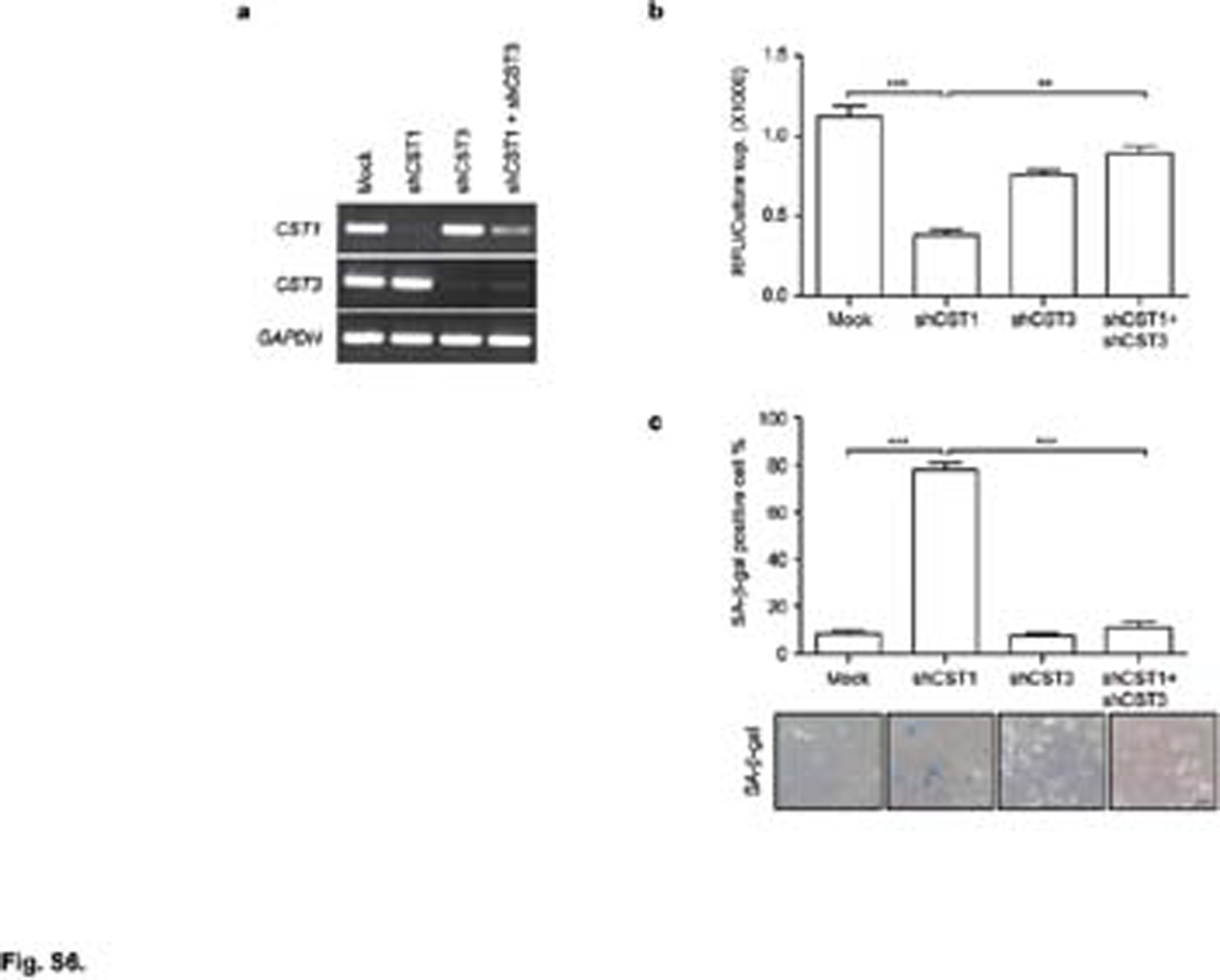

Supplement: Supplementary Figure 6 [file cddis2017153x7.tif]

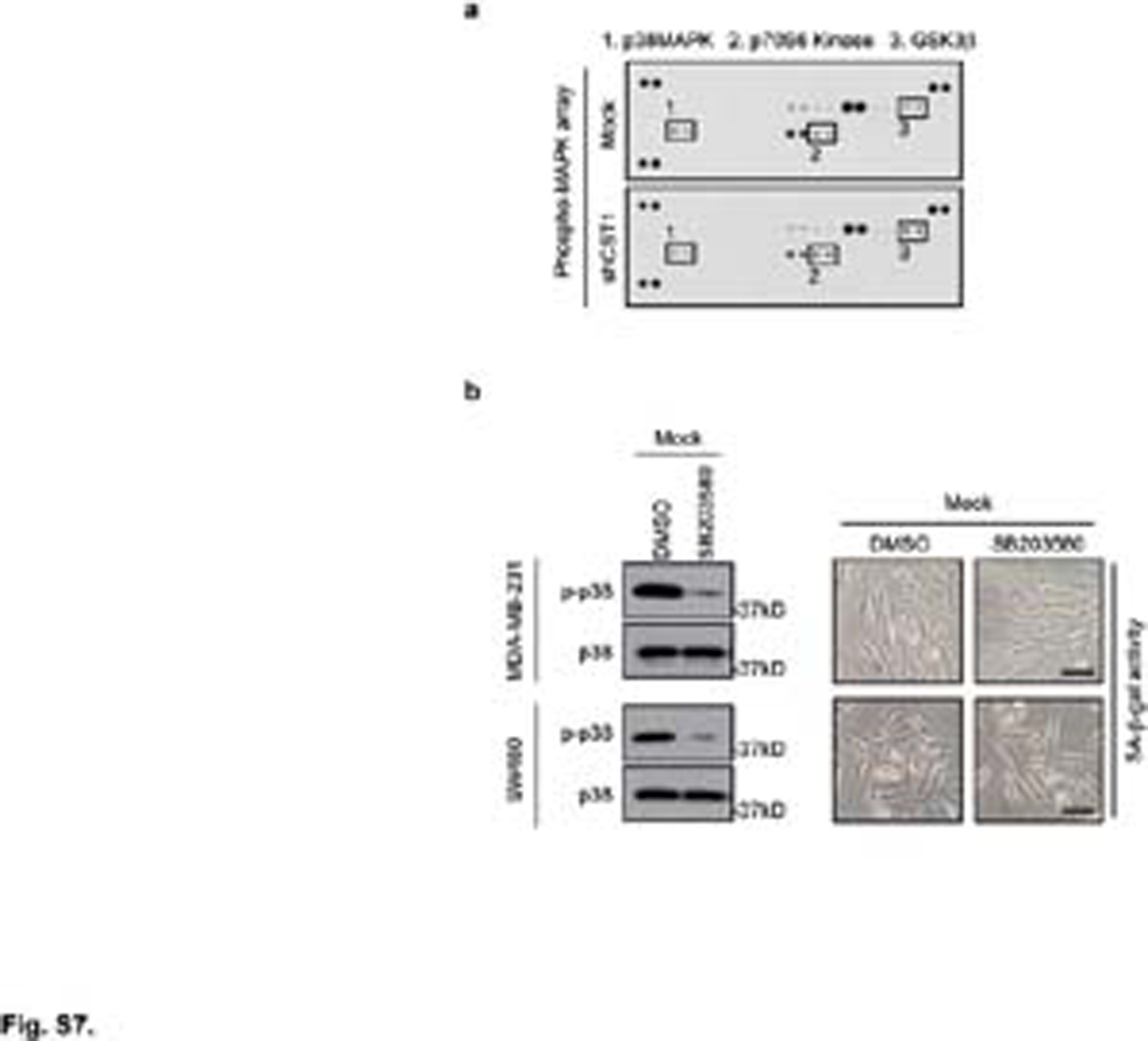

Supplement: Supplementary Figure 7 [file cddis2017153x8.tif]

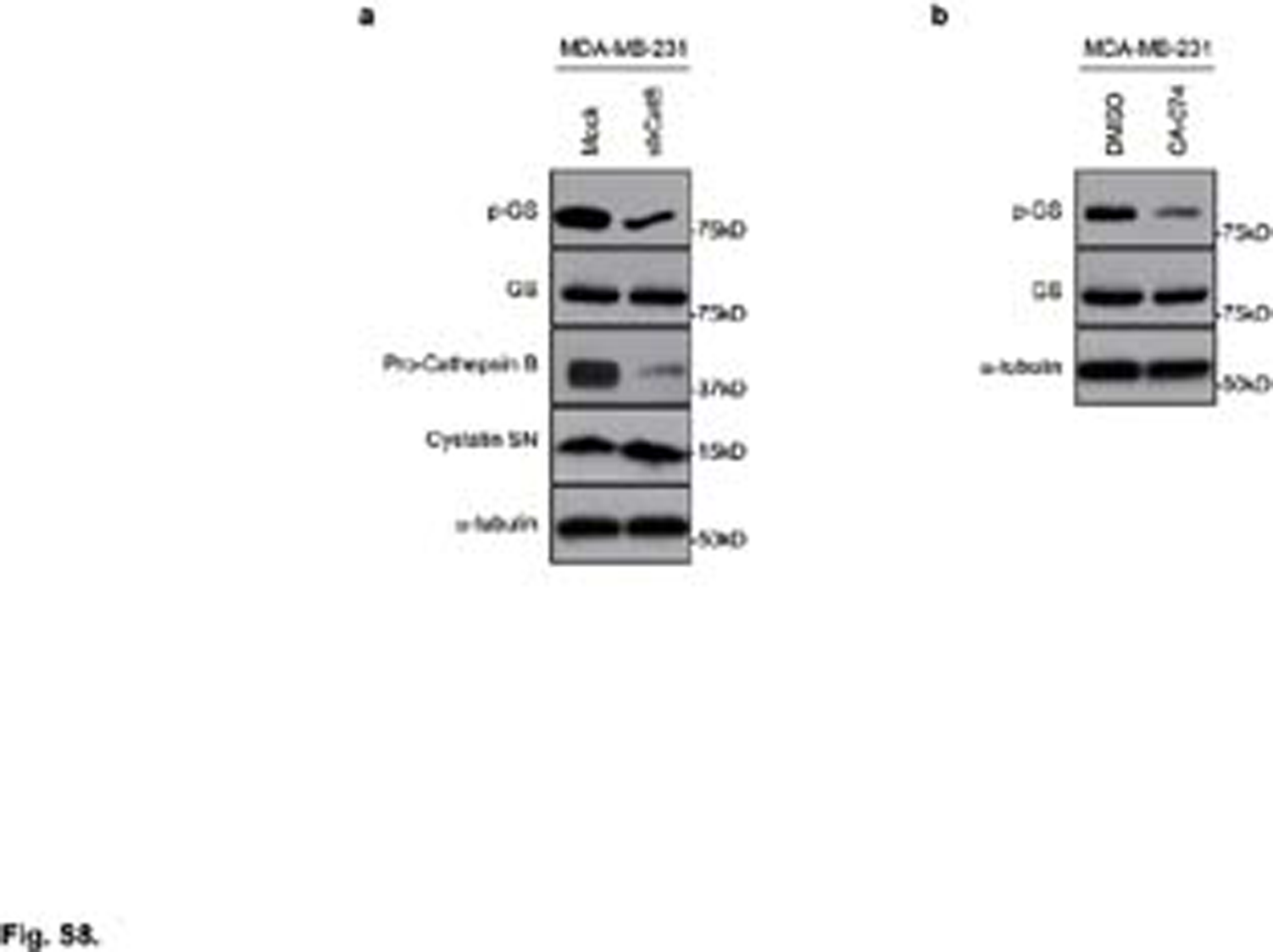

Supplement: Supplementary Figure 8 [file cddis2017153x9.tif]

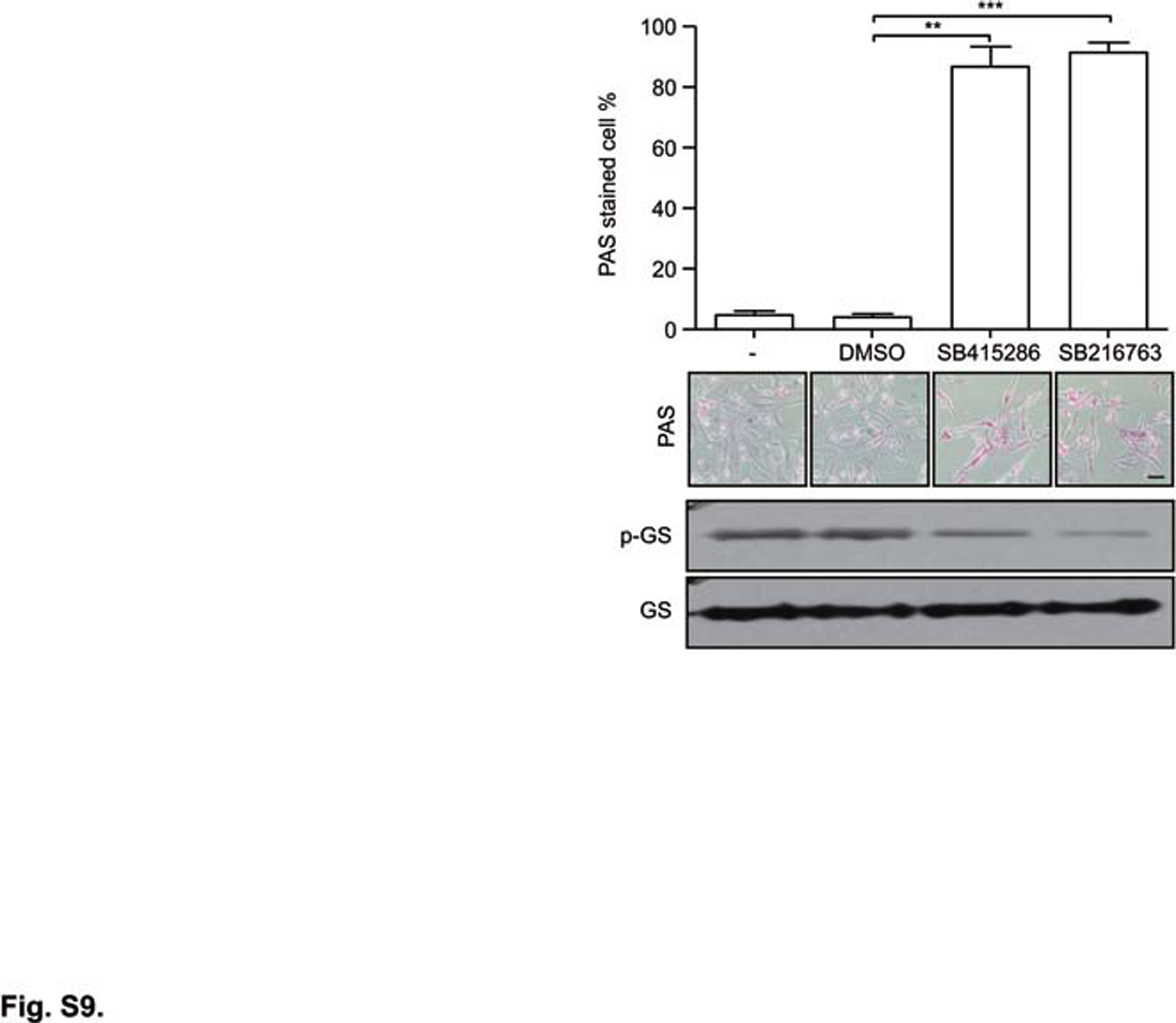

Supplement: Supplementary Figure 9 [file cddis2017153x10.tif]
